# The Pattern of Adipose Tissue Accumulation during Early Infancy Provides an Environment for the Development of Dengue Hemorrhagic Fever

**DOI:** 10.1371/journal.pntd.0004267

**Published:** 2015-12-04

**Authors:** Daniel H. Libraty, Pengyan Wang, Zhiru Guo, Venelle Bigcas, Job D. Brion, Rosario Z. Capeding

**Affiliations:** 1 Division of Infectious Diseases and Immunology, Department of Medicine, University of Massachusetts Medical School, Worcester, Massachusetts, United States of America; 2 College of Animal Sciences and Technology, Shihezi University, Shihezi, China; 3 Department of Medicine, Research Institute for Tropical Medicine, Metro Manila, Philippines; 4 San Pablo City Health Department, San Pablo, Laguna, Philippines; Tropical Medicine Institute Pedro Kourí (IPK), CUBA

## Abstract

**Background:**

Dengue is the most prevalent arthropod-borne viral illness in humans with half of the world’s population at risk. During early infancy, severe dengue can develop after a primary dengue virus infection. There has been a clinical observation that severe dengue during the first year of life is seen only in chubby infants.

**Methodology/Principal Findings:**

We examined the associations between the development of severe dengue and adipose tissue accumulation patterns during the first year of life in a prospective observational clinical study of infants and dengue virus infections. We found that adipose tissue contains two potential targets for dengue virus infection and production- adipocytes and adipose tissue macrophages. During the first year of life, total body adiposity and visceral adipose tissue stores were at their highest levels in early infancy. Early infancy was also characterized by a relative decrease in alternatively activated (anti-inflammatory) macrophages, and a relative increase in circulating pro-inflammatory cytokines.

**Conclusions/Significance:**

The data has been used to propose a model where the adipose tissue accumulation pattern and pro-inflammatory environment during early infancy provide the conditions for the potential development of severe dengue in immune-susceptible infants.

## Author Summary

Dengue is the most prevalent arthropod-borne viral illness in humans with half of the world’s population at risk. During early infancy, severe dengue can develop after a primary dengue virus infection. There has been a clinical observation that severe dengue during the first year of life is seen only in chubby infants. We therefore examined the associations between the development of severe dengue and adipose tissue accumulation patterns during the first year of life in a prospective observational clinical study of infants and dengue virus infections. We found that adipose tissue contains two potential targets for dengue virus infection and production- adipocytes and adipose tissue macrophages. During the first year of life, total body adiposity and visceral adipose tissue stores were at their highest levels in early infancy. Early infancy was also characterized by a relative decrease in alternatively activated (anti-inflammatory) macrophages, and a relative increase in circulating pro-inflammatory cytokines. The accumulation of visceral adipose tissue, the relative decrease in alternatively activated macrophages, and the relative increase in circulating pro-inflammatory cytokines during early infancy provide the environment for the development of severe dengue in immune-susceptible infants.

## Introduction

Dengue is the most prevalent arthropod-borne viral illness in humans with half of the world’s population at risk. The global burden of symptomatic dengue is on the order of 100 million cases/year [1]. The dengue viruses (DENVs) are single-stranded, positive-sense, RNA-containing enveloped viruses belonging to the *Flavivirus* genus within the Flaviviridae family [2]. There are four serotypes of DENVs (DENV1-4). DENV infections produce a wide spectrum of clinical illness. It ranges from asymptomatic or mild illness, to classic dengue fever (DF), to a severe and potentially life threatening disease, dengue hemorrhagic fever (DHF)/dengue shock syndrome (DSS) (severe dengue). The majority of severe dengue is characterized by a transient vascular leakage, and is associated with high viral loads and a pro-inflammatory cytokine storm [3,4]. Most severe dengue is seen in older children and adults with a heterologous (secondary) DENV infection [3,5]. However, DHF/DSS is also seen during early infancy among infants with a primary DENV infection [3,6].

There has been a clinical observation that DHF/DSS during the first year of life is seen only in chubby infants. We therefore examined the associations between the development of infant DHF/DSS and adipose tissue accumulation patterns during the first year of life in a prospective observational clinical study of infants and DENV infections that we have been conducting in San Pablo, Laguna, Philippines [7,8]. Total body adiposity is comprised of visceral adipose tissue and subcutaneous adipose tissue [9]. We found that adipose tissue contains two potential targets for DENV infection and production- adipocytes and adipose tissue macrophages. During the first year of life, total body adiposity and visceral adipose tissue stores were at their highest levels in early infancy. Early infancy was also characterized by a relative decrease in alternatively activated (anti-inflammatory) macrophages, and a relative increase in circulating pro-inflammatory cytokines. The data has been used to propose a model where the adipose tissue accumulation pattern and pro-inflammatory environment during early infancy provide the conditions for the potential development of DHF/DSS in immune-susceptible infants.

## Methods

### Ethics statement

The clinical study protocol was approved by the institutional review boards of the Research Institute for Tropical Medicine, Philippines, and the University of Massachusetts Medical School. Mothers and their healthy infants were recruited and enrolled after providing written informed consent.

### Viruses, cells

The prototype DENV2 strain NGC was used for the adipocyte infection experiments. De-identified matched pairs of visceral (omental) and subcutaneous pre-adipocytes were purchased from Zen-Bio (Research Triangle Park, NC).

### DENV infection of adipocytes

Pre-adipocytes were seeded into wells and terminally differentiated into mature adipocytes, according to the manufacturer’s instructions. The adipocytes were adsorbed with DENV2 NGC at a multiplicity of infection (MOI) = 3 x 2 h, washed x 3, and supernatant was collected for DENV quantitative (q)RT-PCR from days 0–4. Experiments were performed in quadruplicate. Viral RNA was extracted from the supernatants (Qiagen), and DENV qRT-PCR was performed, as previously described [10].

### Clinical study

Details regarding the study protocol have been previously described [8]. In brief, study enrollment began in 2007 in San Pablo, Laguna, Philippines. Healthy infants and their mothers were enrolled when the infant was around 2 months old. A subset of infants returned approximately every 2 months over the first year of life for study visits. Blood samples, clinical, and epidemiological information were collected at the study visits.

We conducted surveillance year-round for hospitalized acute febrile illnesses in study infants across the seven hospitals serving San Pablo. During the rainy season (June-November), mothers were also encouraged to bring their infants to the San Pablo City Health Office for evaluation of outpatient febrile illnesses.

A DENV infection was identified in febrile infants by serotype-specific RT-PCR in acute-phase sera [11] and DENV IgM/IgG ELISA [12] in paired acute and convalescent phase sera. Primary or secondary DENV infections were identified by previously established serologic criteria for the paired IgM/IgG ELISA results [12]

### Anthropometric measurements

At each study visit, weight was measured to the nearest tenth of a kg, length was measured to the nearest tenth of a cm, and subscapular skinfold thickness was measured to the nearest mm by calipers. Body mass index (BMI) was calculated as weight (kg)/(length (m))^2^, and was used as an estimate of total body adiposity. The estimated subcutaneous adipose tissue mass was calculated as the subscapular skinfold thickness (mm) x body surface area (BSA)(m^2^) (√(length (cm))x(weight (kg))/3600). The estimated visceral adipose tissue mass was calculated as BMI—estimated subcutaneous adipose tissue mass.

### Multiplex soluble (s)CD163 and cytokine assays

sCD163, tumor necrosis factor-α (TNF-α), interleukin-1β (IL-1β), and IL-6 levels were measured in longitudinally collected sera over the first year of life from 176 healthy infants using a multiplex assay, according to the manufacturer’s instructions (Bio-Plex, Bio-Rad).

## Results

### DENV infects adipocytes

**Fig 1:**
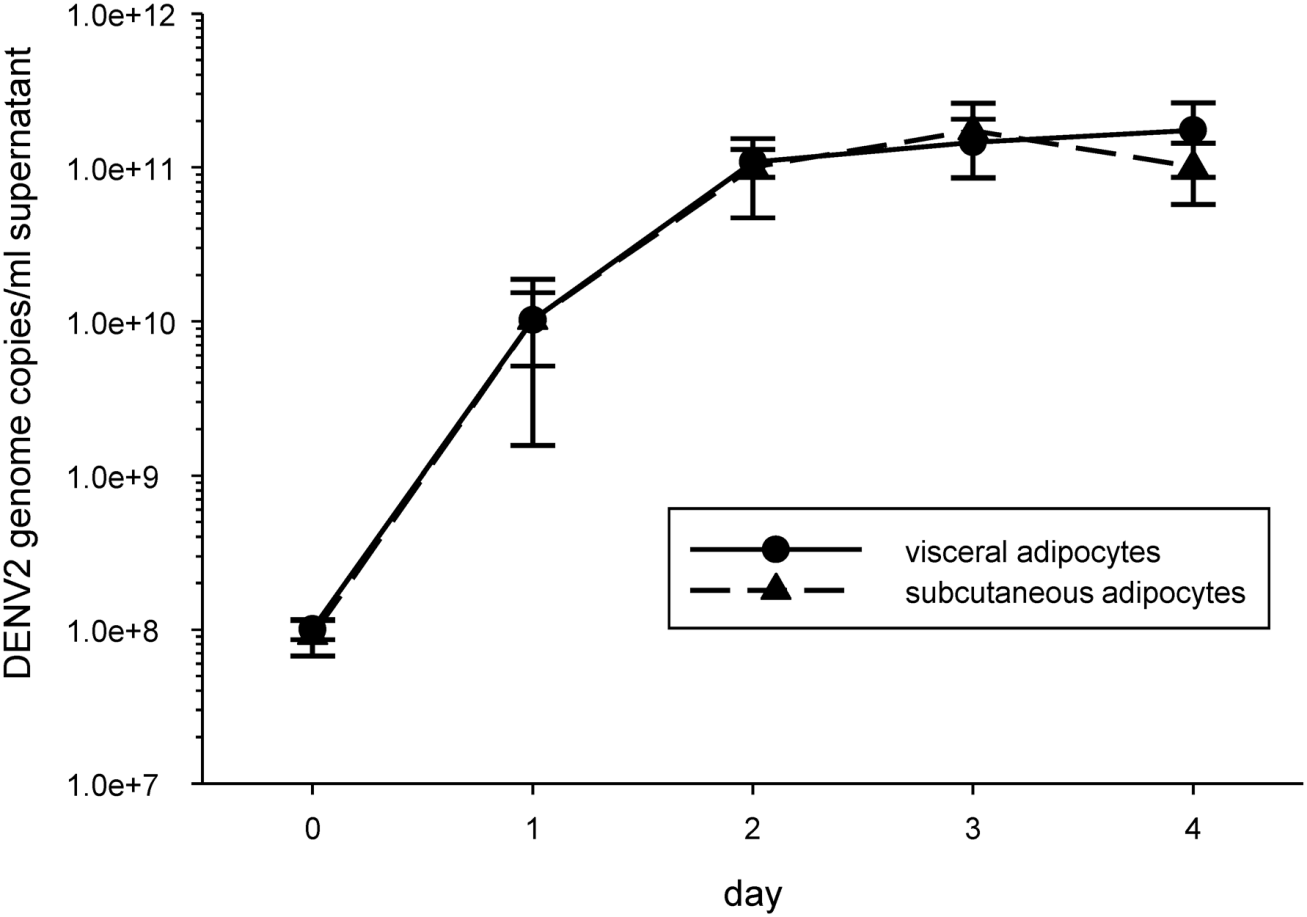
Dengue virus type 2 (DENV2) productively infects visceral and subcutaneous adipocytes. Matched pairs of visceral and subcutaneous pre-adipocytes from 3 donors were terminally differentiated to mature adipocytes and infected with DENV2 NGC at a multiplicity of infection (MOI) = 3. Cell culture supernatants were collected each day, between days 0–4, for DENV2 quantitative (q)RT-PCR. Symbols and error bars are mean±SEM.

Paired visceral and subcutaneous pre-adipocytes were differentiated to mature adipocytes and infected with DENV2 strain NGC at MOI = 3. 60–80% of the cells in each well were mature adipocytes. DENV2 NGC was able to productively infect visceral and subcutaneous adipocytes equally well (Fig 1). Adipose tissue consists of approximately 80% adipocytes and 20% stromal cells. The majority of stromal cells are macrophages. Thus, adipose tissue contains two potential target cells for DENV infection and production- adipocytes and adipose tissue macrophages [3,13].

### Infant DHF/DSS is seen when visceral adipose tissue stores are around their highest levels in the first year of life

**Fig 2:**
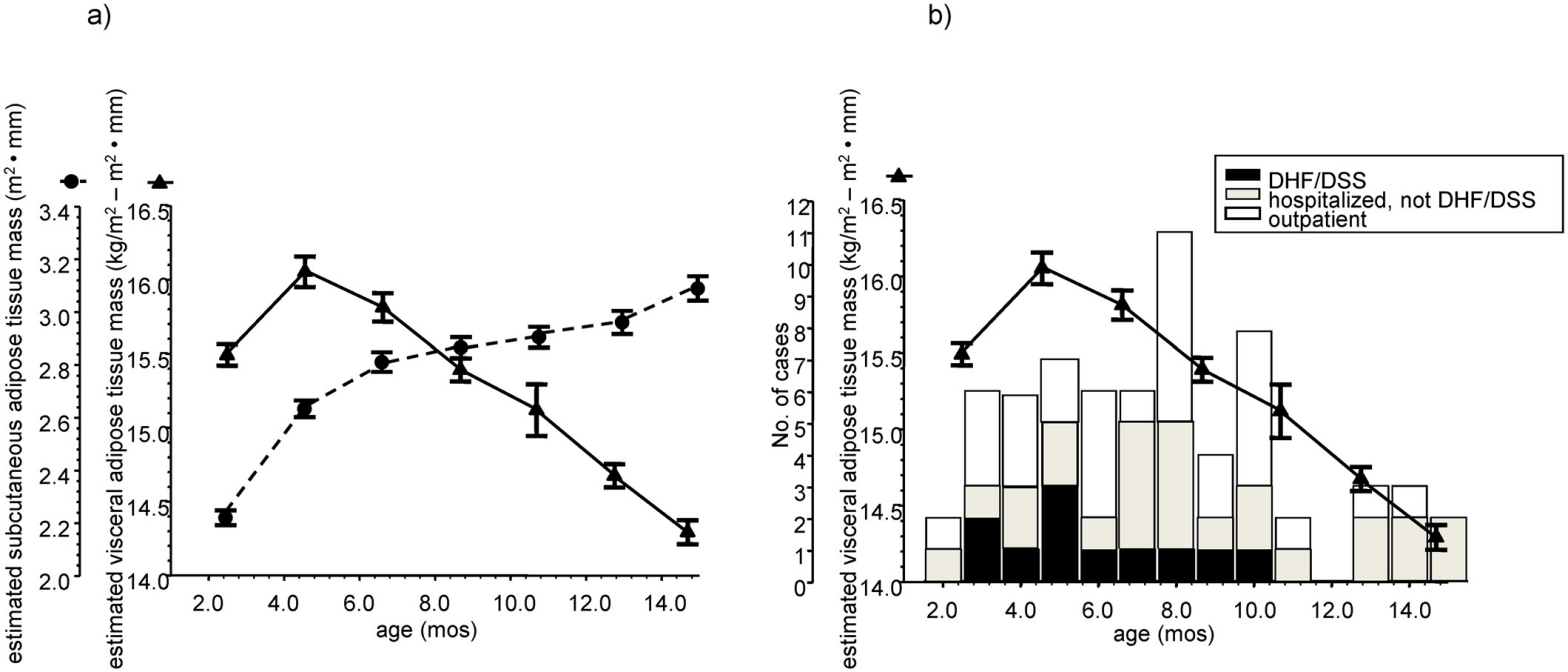
**(a) Longitudinal changes in estimated visceral adipose tissue mass and estimated subcutaneous adipose tissue mass in 427 healthy infants over the first year of life.** Equations used to estimate the visceral and subcutaneous adipose tissue stores are described in the Methods section. Symbols and error bars are mean±SEM plotted at the mean ages of the study visits. **(b) Longitudinal changes in visceral adipose tissue mass from figure 2a, and the ages of infants sustaining a primary dengue virus infection with their clinical disease severity classification.** DHF/DSS = dengue hemorrhagic fever/dengue shock syndrome. Symbols and error bars are mean±SEM plotted at the mean ages of the study visits.

We performed longitudinal anthropometric measurements in 427 healthy infants over the first year of life. BMI was used as an estimate of total body adiposity, and BMI peaked between the ages of 4–7 months old (S1 Fig). Subscapular skinfold thickness was used as a measure of subcutaneous fat. The equations used to estimate the subcutaneous and visceral adipose tissue stores in the infants are described in the Methods section. Over the first year of life, the estimated subcutaneous adipose tissue mass gradually increased, while the estimated visceral adipose tissue mass reached its highest level around ages 4–7 months old, and then decreased (Fig 2a). Primary DENV infections in infants that led to DHF/DSS clustered between the ages where visceral adipose tissue stores were around their highest levels (Fig 2b).

### Circulating levels of sCD163 were around their nadir when visceral adipose tissue stores were around their highest levels in the first year of life

**Fig 3:**
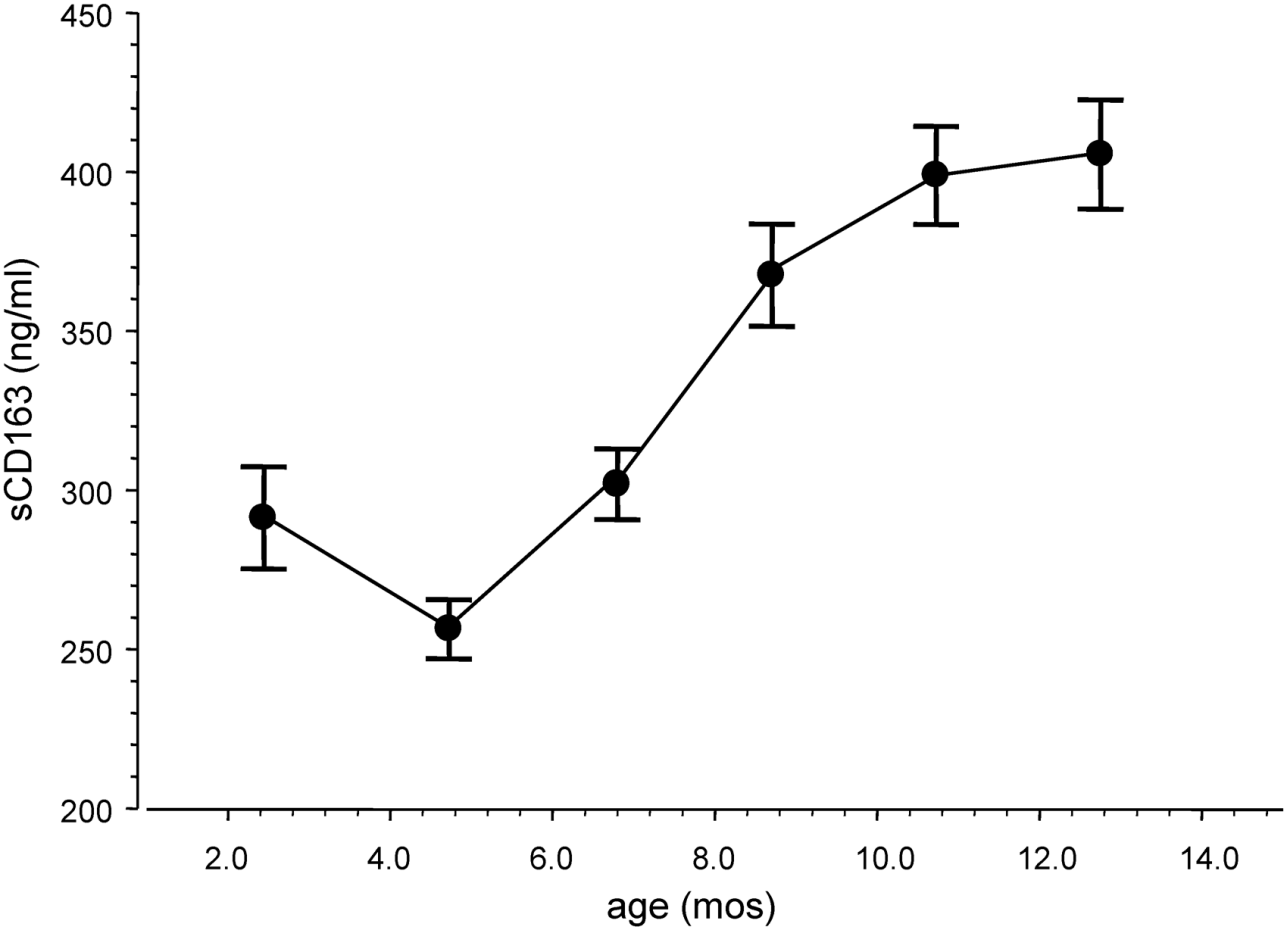
Longitudinal changes in the serum levels of soluble (s)CD163 in 176 healthy infants over the first year of life. Symbols and error bars are mean±SEM plotted at the mean ages of the study visits.

We measured in a longitudinal manner the serum levels of sCD163 in 176 healthy infants over the first year of life. The mean circulating levels of sCD163 decreased after birth, reached their lowest levels between ages 4–7 months old, and then gradually increased (Fig 3). sCD163 is a macrophage activation marker that can be increased by a pro-inflammatory state or an increase in alternatively activated macrophages [14]. During infancy, the nadir of circulating sCD163 levels occurred when circulating pro-inflammatory cytokines were at their highest levels. This suggests that circulating sCD163 levels during infancy reflected the alternative activation state of macrophages.

### Circulating levels of pro-inflammatory cytokines were at their highest levels in the first year of life between ages 4–7 months old

**Fig 4:**
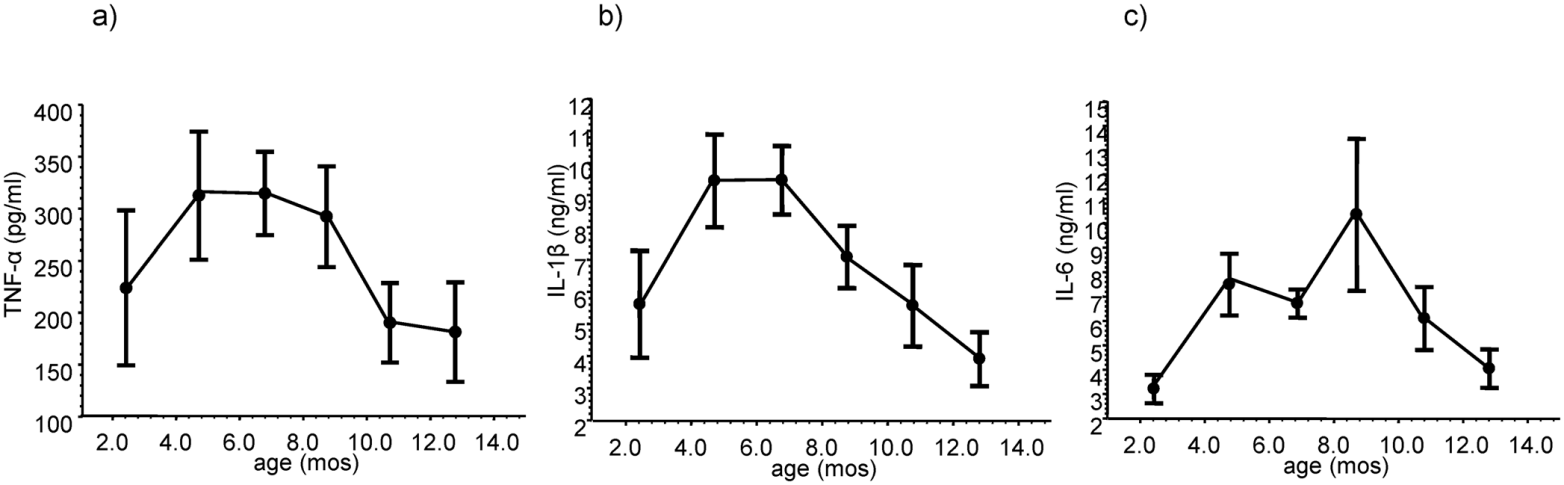
Longitudinal changes in the serum levels of (a) tumor necrosis factor-α (TNF-α), (b) interleukin-1β (IL-1β), and (c) IL-6 in 176 healthy infants over the first year of life. Symbols and error bars are mean±SEM plotted at the mean ages of the study visits.

We measured in a longitudinal manner over the first year of life the serum levels of TNF-α, IL-1β, and IL-6 in the same 176 infants with sCD163 determinations. The mean circulating levels of these pro-inflammatory cytokines increased after birth, reached their highest levels between the ages of 4–7 months old, and then decreased (Fig 4).

## Discussion

In adipose tissue, DENV can productively infect adipocytes and macrophages. We observed that infants who developed unambiguous DHF/DSS after a primary DENV infection clustered between the ages of 3–10 months old. In this age range, estimated visceral adipose tissue mass was also around its highest level during the first year of life. Mean circulating levels of a marker for alternatively activated macrophages were relatively decreased during this time period, and mean circulating levels of pro-inflammatory cytokines were relatively increased. This data has led us to propose a model for infant DHF/DSS risk factors that explains the clinical observation that DHF/DSS during the first year of life is seen only in chubby infants.

First, we propose that adipose tissue (specifically adipocytes and adipose tissue macrophages) is an important source of DENV production. During the first year of life, severe dengue (DHF/DSS) can develop with a primary DENV infection when total body adiposity and visceral adipose tissue stores are around their highest levels. When visceral adipose tissue mass is around its highest level in early infancy, there is also a relative decrease in alternatively activated (anti-inflammatory) macrophages [15] and there exists a pro-inflammatory cytokine environment. When infants become immune-susceptible (maternally-acquired anti-DENV antibody titers fall below protective levels), these conditions provide an environment where there can be high viral loads with the development of a pro-inflammatory cytokine storm- the risk factors for developing DHF/DSS. Childhood and adult obesity are associated with increased visceral adipose tissue stores, macrophage infiltration of the visceral adipose tissue, a relative paucity of alternatively activated visceral adipose tissue macrophages, and a pro-inflammatory cytokine environment [16–18]. We suggest that a similar pattern of adipose tissue accumulation and macrophage infiltration and activation is seen during early infancy, and this pattern creates the conditions for the potential development of severe dengue in immune-susceptible infants after a primary DENV infection.

## Supporting Information

S1 Fig Longitudinal changes in body mass index (BMI) (weight (kg)/height (m)^2^) in 427 healthy infants over the first year of life. Symbols and error bars are mean±SEM plotted at the mean ages of the study visits.
(TIFF)
